# Vaccine-Induced Protection in Two Murine Models of Invasive Pulmonary Aspergillosis

**DOI:** 10.3389/fimmu.2021.670578

**Published:** 2021-05-18

**Authors:** Emily Rayens, Whitney Rabacal, S. Earl Kang, Brandi N. Celia, Michelle Momany, Karen A. Norris

**Affiliations:** ^1^ Center for Vaccines and Immunology, University of Georgia, Athens, GA, United States; ^2^ Department of Infectious Diseases, University of Georgia, Athens, GA, United States; ^3^ Department of Plant Biology, University of Georgia, Athens, GA, United States

**Keywords:** aspergillus, invasive pulmonary aspergillosis, fungal vaccines, FK506 (tacrolimus), immunocompromised patients

## Abstract

Life-threatening, invasive fungal infections (IFIs) cause over 1.5 million deaths worldwide and are a major public health concern with high mortality rates even with medical treatment. Infections with the opportunistic fungal pathogen, *Aspergillus fumigatus* are among the most common. Despite the growing clinical need, there are no licensed vaccines for IFIs. Here we evaluated the immunogenicity and protective efficacy of an *A. fumigatus* recombinant protein vaccine candidate, AF.KEX1, in experimental murine models of drug-induced immunosuppression. Immunization of healthy mice with AF.KEX1 and adjuvant induced a robust immune response. Following AF.KEX1 or sham immunization, mice were immunosuppressed by treatment with either cortisone acetate or hydrocortisone and the calcineurin inhibitor, tacrolimus. To test vaccine efficacy, immunosuppressed mice were intranasally challenged with *A. fumigatus* conidia (Af293) and weight and body temperature were monitored for 10 days. At study termination, organism burden in the lungs was evaluated by quantitative PCR and Gomori’s methanamine silver staining. In both models of immunosuppression, AF.KEX1 vaccinated mice experienced decreased rates of mortality and significantly lower lung organism burden compared to non-vaccinated controls. The lung fungal burden was inversely correlated with the peak anti-AF.KEX1 IgG titer achieved following vaccination. These studies provide the basis for further evaluation of a novel vaccine strategy to protect individuals at risk of invasive aspergillosis due to immunosuppressive treatments.

## Introduction

Globally, *Aspergillus* infections are estimated to affect over 3 million people and contribute to nearly 500,000 deaths per year (1, 2). There are several clinical manifestations of *Aspergillus* exposure. *Aspergillus* colonization is a major cause of allergic diseases, which can contribute to severity and exacerbations of chronic pulmonary diseases such as asthma and cystic fibrosis. Additionally, fungal sensitization in severe asthma may affect over 6 million people (3). The most severe infection due to *Aspergillus* is invasive pulmonary aspergillosis (IPA) which is one of the most frequent, life-threatening infections of immunosuppressed individuals. The incidence of IPA is increasing, largely due to the increases in patient populations receiving immunosuppressive medications for autoimmune disease, post-transplantation management, cancer chemotherapy and other inflammatory diseases. Prospective studies of transplant recipients performed during 2001-2006 found that IPA was the most common type of fungal infection among stem cell transplant recipients (HSCT) (4) and was the second-most common type of fungal infection among solid organ transplant recipients (5). In a study of US intensive care units, IPA was reported to be the fourth most common diagnosis that likely lead to death (1). Despite the increases in morbidity and mortality due to IPA and increasing concerns for an emerging trend in azole resistance (6), there are no anti-fungal vaccines approved for clinical use and few options have been presented in the field as candidates (7). Thus, there is a critical need for alternative prophylactic and therapeutic options that are safer and more effective at preventing and treating IPA.

Our lab has previously investigated the host immune response to other opportunistic fungal pathogens, including *Pneumocystis jirovecii* (previously *Pneumocystis carinii*) and identified a vaccine candidate, Pc.KEX1, based on a highly conserved, internal region of the *Pneumocystis* endoprotease Kexin, which was sub-cloned and expressed in *E. coli* (8). In an experimental non-human primate model of HIV-associated *Pneumocystis* pneumonia (PCP), we demonstrated that immunization of healthy animals with recombinant Pc KEX1 protected against PCP following simian immunodeficiency virus (SIV)-induced immunosuppression and *Pneumocystis* challenge (9). The *Pneumocystis* KEX1 sequence is highly conserved among pathogenic fungi, including *A. fumigatus* (Norris, K., unpublished). In the present study, we cloned the homologous KEX1 region from *A. fumigatus* and evaluated the immune response to recombinant AF.KEX1 in mice experimentally infected with *A. fumigatus* and the protective efficacy of immunization with AF.KEX1 against IPA in immunocompromised mice.

## Materials and Methods

### Vaccine Construction and Purification

**Figure 1:**
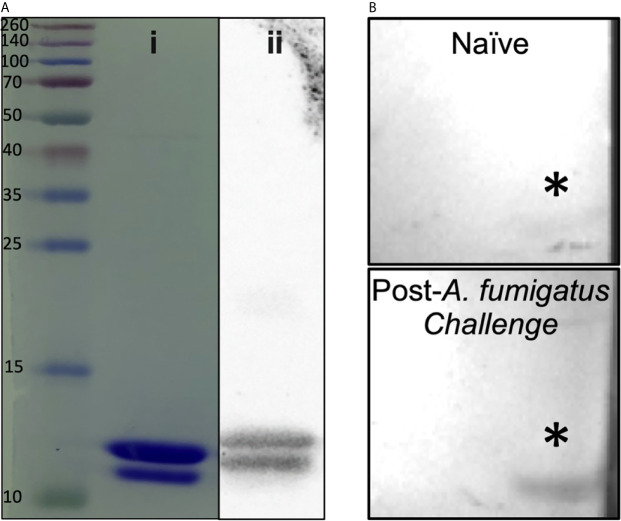
AF.KEX1 purification and humoral responses following *A. fumigatus* challenge. **(A)** Purified AF.KEX1 protein by (*i*) Coomassie and (*ii*) anti-histidine Western blot. **(B)** Western blots of recombinant AF.KEX1 using plasma from a single mouse prior to and following *A. fumigatus* challenge. AF.KEX1 bands are indicated by *.

A 90 amino acid fragment of *Aspergillus fumigatus* KexB (AF.KEX1, reference sequence XP_751534.1; residue numbers 300-389) was cloned into the pET28b(+) expression vector (Novagen) in *Escherichia coli* BL21(DE3) pLysS (ThermoFisher, Scientific). Briefly, AF.KEX1 expression was induced in *E. coli;* cells were lysed and centrifuged, and supernatant was purified by affinity chromatography. Purified protein was run on SDS-PAGE gels (**Figure 1Ai**), transferred to nitrocellulose, blocked, and incubated with 6x-His tag monoclonal antibody (**Figure 1Aii**), as well as with plasma obtained prior to and following *A. fumigatus* Af293-challenged mice from the present study (**Figure 1B**). Filters were washed, incubated with horseradish peroxidase-conjugated IgG secondary antibody, and developed according to standard protocols. Purified AF.KEX1 was used for immunization and enzyme-linked immunosorbent assay (ELISA).

### Immunization of Mice

As detailed in **Table 1**, 5 CF-1 (Group 1) and 12 BALB/c (Group 5) mice were immunized subcutaneously at the base of the tail with 40-50ug AF.KEX1 prepared 1:1 with a water:squalene adjuvant TiterMax (Sigma Aldrich) according to the manufacturer’s guidelines. An additional 7 CF-1 (Group 2) and 13 BALB/c (Group 6) were sham immunized with PBS and TiterMax. A further 9 CF-1 mice were vaccinated and subsequently boosted with 50ug AF.KEX1 prepared 1:1 with TiterMax to evaluate the need for a vaccine boost (Group 3). A final group of 10 CF-1 received no vaccination (Group 4). Blood was collected at day of immunization, and 14, 21, and 28 days following. Plasma samples were stored at - 80°C.

**Table 1:** Vaccine Groups.

Immunosuppression	Group	n (Mice)	n (Immunizations)	Vaccine
Steroid Immunosuppression	1	5	1	AF.KEX1+TiterMax
	2	7	1	PBS+TiterMax
	3	9	2	AF.KEX1+TiterMax
	4	10	0	No Vaccine
Tacrolimus/Hydrocortisone Immunosuppression	5	12	1	AF.KEX1+TiterMax
	6	13	1	PBS+TiterMax

### Steroid Immunosuppression Model

32 CF-1 female mice (Charles River Laboratories) were randomly assigned to four cohorts for immunization and challenge, detailed in **Table 1**. 28 days following the final vaccination, all CF-1 mice began an immunosuppressive regimen of 2.5 mg cortisone acetate per mouse in PBS with 0.5% methylcellulose and 0.01% Tween-80 injected subcutaneously (10). This regimen was administered for six days, during which time trimethoprim sulfamethoxazole (TMP/SMX) was added to the drinking water to control secondary infections. All studies were approved by the Institutional Animal Care and Use Committee of the University of Georgia.

### Tacrolimus/Hydrocortisone Immunosuppression Model

Male and female BALB/c (Charles River Laboratories) were randomly assigned to vaccine and control groups (**Table 1**). 28 days following the final vaccination, all mice were treated with 125 mg/kg hydrocortisone (Sigma Aldrich) injected subcutaneously every three days and 1 mg/kg tacrolimus (PKC Pharmaceuticals) intraperitoneally every day, as described in a model of solid organ transplantation (11). This regimen began three days prior to challenge and continued for the duration of the study. TMP/SMX was added to the drinking water to prevent bacterial infections.

### ELISA Assays

Microtiter plates (Immunolon 4HBX; Thermo Fisher Scientific) were coated with purified AF.KEX1 at 5 µg/ml in PBS. Heat-inactivated plasma samples were diluted 1:100 in blocking buffer (PBS with 5% nonfat milk) and 1:2 serial dilutions were made to determine endpoint titers. Horseradish peroxidase-conjugated goat anti-mouse IgG1, IgG2a, or IgG antibodies (ThermoFisher Scientific), were diluted according to manufacturer guidelines in PBS-T for use as detection antibodies with TMB substrate (ThermoFisher Scientific). Naive (uninfected, AF.KEX1-negative by antibody titer) mouse plasma was used as a negative control.

### *Aspergillus fumigatus* Challenge and Monitoring

*A. fumigatus* Af293 conidia were maintained on solid 1% glucose minimal media for 72 hours, harvested in 0.01% Tween-20, and counted with a hemocytometer, then diluted in PBS. Mice were briefly anesthetized with isoflurane and inoculated with 5x10 (6) conidia in 40ul PBS *via* intranasal inoculation.

Following challenge, mice were monitored twice daily for changes in weight and temperature, the latter using an infrared thermometer (Sper Scientific). If weight loss greater than 20 percent or body temperature below 29°C was recorded, the animals were euthanized. At six days following challenge, all remaining animals were euthanized with carbon dioxide per UGA Institutional Animal Care and Use Committee guidelines, and lungs were collected for analysis.

### Fungal Burden

At study termination, right lungs were preserved in formalin while the left lungs were frozen in liquid nitrogen. The fixed lung tissue was embedded in paraffin, cut, and stained with Gomori’s modified methenamine silver stain. Five random fields were imaged and fungal burden quantified according to the guidelines provided by Stolz et al. (12).

The frozen lung tissue was lyophilized and homogenized with 0.5 mm disruption glass beads (Research Products International) and three 3 mm steel beads using Geno/Grinder (Fisher Scientific) at 1750 rpm for 30 sec. Total DNA was extracted using a modified CTAB protocol as previously described (13). RNAse treated DNA was used for qPCR with targets and primers as previously described (14) on a Mx3005P real-time PCR system (Agilent Technologies, Inc. Santa Clara, CA, USA). Reaction volume of 50ul with 5ul of diluted DNA and 25ul of 2X PowerUp SYBR Green Master Mix (Applied Biosystems) was used. Thermal cycling conditions were 95C for 10 min; 40 cycles of 95C for 30s and 60C for 1 min. Melt curve analysis was performed at the end of the qPCR reactions to check for primer specificity. Controls without a template were also run. Isolated gDNA and uninfected murine tissue were used as the positive and negative controls, respectively.

### Statistical Analysis

All statistical analyses were performed using GraphPad Prism (GraphPad Software, La Jolla, CA). Differences in post-immunization anti-AF.KEX1 antibody titer were analyzed using Mann-Whitney U tests. Mantel-Cox test was used to analyze the survival curves following *A. fumigatus* challenge. In the steroid immunosuppression model, Groups 1 and 3 were combined for survival analysis against 2 and 4, given that there was no significant difference in AF.KEX1 IgG titer within the pairs of groups. Differences in fungal burden by both GMS staining and qPCR were analyzed by Mann-Whitney U tests. The correlation between anti-AF.KEX1 antibody titer and terminal fungal burden was determined by Spearman correlation.

## Results

### Humoral Immune Responses to AF.KEX1

**Figure 1A** demonstrates purity achieved from AF.KEX1 protein preparation. Based on the immunogenicity and protective efficacy of the conserved KEX1 peptide in *Pneumocystis* infection (9), we first examined whether anti-AF.KEX1 antibodies are elicited during experimental *Aspergillus* infection. Western blot analysis of recombinant AF.KEX1 was performed with plasma obtained pre- and post-*Aspergillus* Af293 infection of cortisone-immunosuppressed CF-1 mice (**Figure 1B**). These results demonstrate that antibodies recognizing AF.KEX1 are naturally generated during experimental *A. fumigatus* infection in immunocompromised mice.

**Figure 2:**
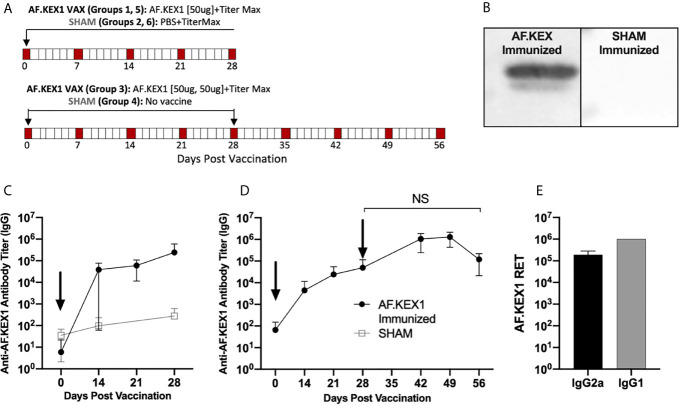
AF.KEX1-specific humoral responses following *A. fumigatus* challenge and AF.KEX1 immunization in mice. **(A)** Schematic of vaccinations for all mice. **(B)** Western blots of recombinant AF.KEX1 protein using plasma from mice 28 days post vaccination with AF.KEX1+TiterMax or PBS+TiterMax. **(C)** Mean plasma AF.KEX1-specific immunoglobulin G (IgG) titer, as determined by enzyme-linked immunosorbent assay of a single immunization or **(D)** two immunizations with AF.KEX1+TiterMax and PBS+TiterMax. **(E)** IgG2a (Th1) and IgG1 (Th2) antibody levels in pooled plasma from AF.KEX1+TiterMax-immunized mice. Vaccinations are indicated with arrows. Mann-Whitney rank tests were performed to compare data baseline to data and all post-vaccination timepoints were p <.0001, compared with baseline. NS, not significant.

**Figure 2A** describes vaccination scheme of healthy mice with recombinant AF.KEX1 emulsified with adjuvant (Titermax) or PBS and adjuvant. Anti-AF.KEX1 antibody titers significantly increased following vaccination, peaking at 28 days post vaccination (mean reciprocal endpoint titer (RET) =2.44x10 (5)), while no significant change was observed in the sham cohorts (**Figure 2C**). These results were further corroborated by Western blot of AF.KEX1 protein, which found that antibody recognition of AF.KEX1 is increased in mice vaccinated with the recombinant protein compared to the sham cohort (**Figure 2B**). We further examined the effect of an AF.KEX1 boost but found that while a higher peak titer was achieved, there was no significant difference in anti-AF.KEX1 IgG titer at 28 days post boost from that achieved at 28 days following initial vaccination (**Figure 2D**). Pooled plasma from AF.KEX1+TiterMax-immunized mice was analyzed for IgG1 and IgG2a antibody levels by ELISA. Immunization resulted in a balanced IgG1 and IgG2a response (**Figure 2E**).

### Humoral AF.KEX1 Vaccination Reduces *Aspergillus*-Associated Morbidity and Mortality in Tacrolimus Immunosuppression Model

**Figure 3:**
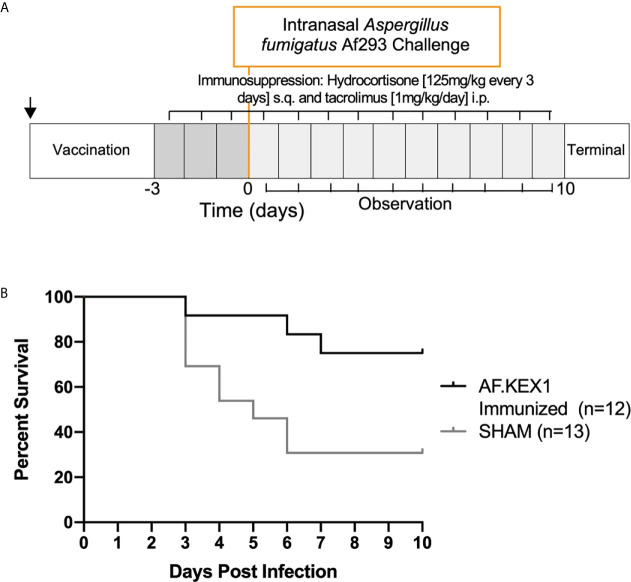
Invasive pulmonary aspergillosis in a tacrolimus/hydrocortisone immunosuppression model. **(A)** Challenge study design. **(B)** Survival curve of AF.KEX1-immunized animals, compared with SHAM. AF.KEX1-immunized animals were significantly protected from developing aspergillosis while immunosuppressed with tacrolimus and hydrocortisone, compared with sham-immunized controls (*p* = .0183, by Mantel-Cox test). Immunization is indicated by arrow.

The experimental design for this study is shown in **Figure 3**, based on a previously reported model of murine aspergillosis (11). Following a single vaccination with either AF.KEX1+Titermax or PBS+TiterMax, BALB/c mice were immunosuppressed for three days with the calcineurin inhibitor tacrolimus and hydrocortisone, based upon the immunosuppression drug regimen of a solid organ transplant recipient (11).

**Figure 4:**
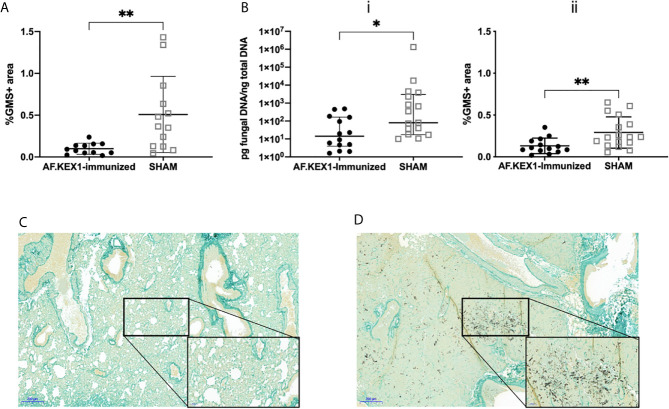
Lung fungal burden following *A. fumigatus* challenge. **(A)** Quantification of fungal burden in the lungs of tacrolimus/hydrocortisone-immunosuppressed mice by %GMS+ area. **(B)** Quantification of the fungal burden in the lungs of cortisone acetate-immunosuppressed mice using (i) qPCR of fungal DNA and (ii) Gomori’s methenamine silver (GMS) staining.** (C)** GMS staining of the lungs of cortisone acetate-immunosuppressed mice following vaccination with AF.KEX1 and Af293 challenge or **(D)** SHAM vaccination and Af293 challenge (20x magnification). Mann-Whitney rank tests were performed to compare cohorts. **p < 0.01, *p < 0.05.

Following intranasal challenge with *A. fumigatus* Af293 conidia (**Figure 3A**), weight and temperature were monitored twice daily for 10 days. Over the observation period, 7 of the 13 sham-immunized cohort were euthanized for aspergillosis-related endpoints while only 2 of the 12 mice vaccinated with AF.KEX1 met the same endpoints (*p* = .0183, **Figure 3B**). The fungal burden in AF.KEX1-vacinated mice was significantly lower compared to the sham-immunized cohort (*p*=0.0023, **Figure 4A**). There were no significant differences in mortality or fungal burden between sexes (*p*=.7551, *p*=.2002).

### AF.KEX1 Vaccination Reduces *Aspergillus*-Associated Mortality and Morbidity in a Steroid Immunosuppression Model

**Figure 5:**
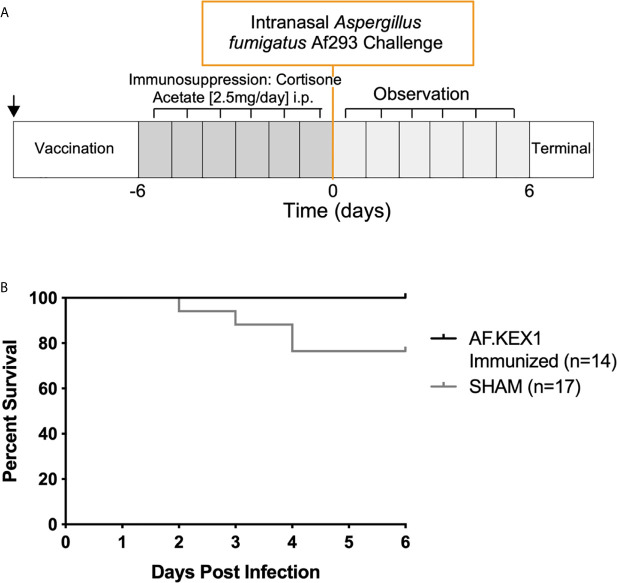
Invasive pulmonary aspergillosis in steroid immunosuppression model. **(A)** Challenge study design. **(B)** Survival curve of AF.KEX1-immunized animals, compared with SHAM. AF.KEX1-immunized animals were significantly protected from developing Aspergillosis when immunosuppressed with cortisone acetate, compared with sham-immunized controls (*p*=.0487, by Mantel-Cox test). Immunization is indicated by arrow.

To test the protective efficacy in an additional model of immunosuppression (10), we immunized CF-1 mice as shown in **Figure 2A**, followed by immunosuppression with cortisone acetate, and challenged with *A. fumigatus* Af293 conidia (**Figure 5**). The mice were monitored twice daily as above. One AF.KEX1-immunized mouse was censured from analysis as mortality occurred unrelated to this study. Over the observation period, 4 of the 17 sham-immunized cohort were euthanized for aspergillosis-related endpoints while there was no mortality in those vaccinated with AF.KEX1 (*p* = .0487). By both qPCR (**Figure 4Bi** *)* and histological quantification (**Figure 4Bii** *)*, fungal burden was significantly decreased in the cohorts immunized with AF.KEX1+TiterMax. Representative images of AF.KEX1- and sham-immunized lung tissue are seen in **Figures 4C, D**, respectively.

### Peak AF.KEX1 IgG Is Correlated With Lung Fungal Burden

**Figure 6:**
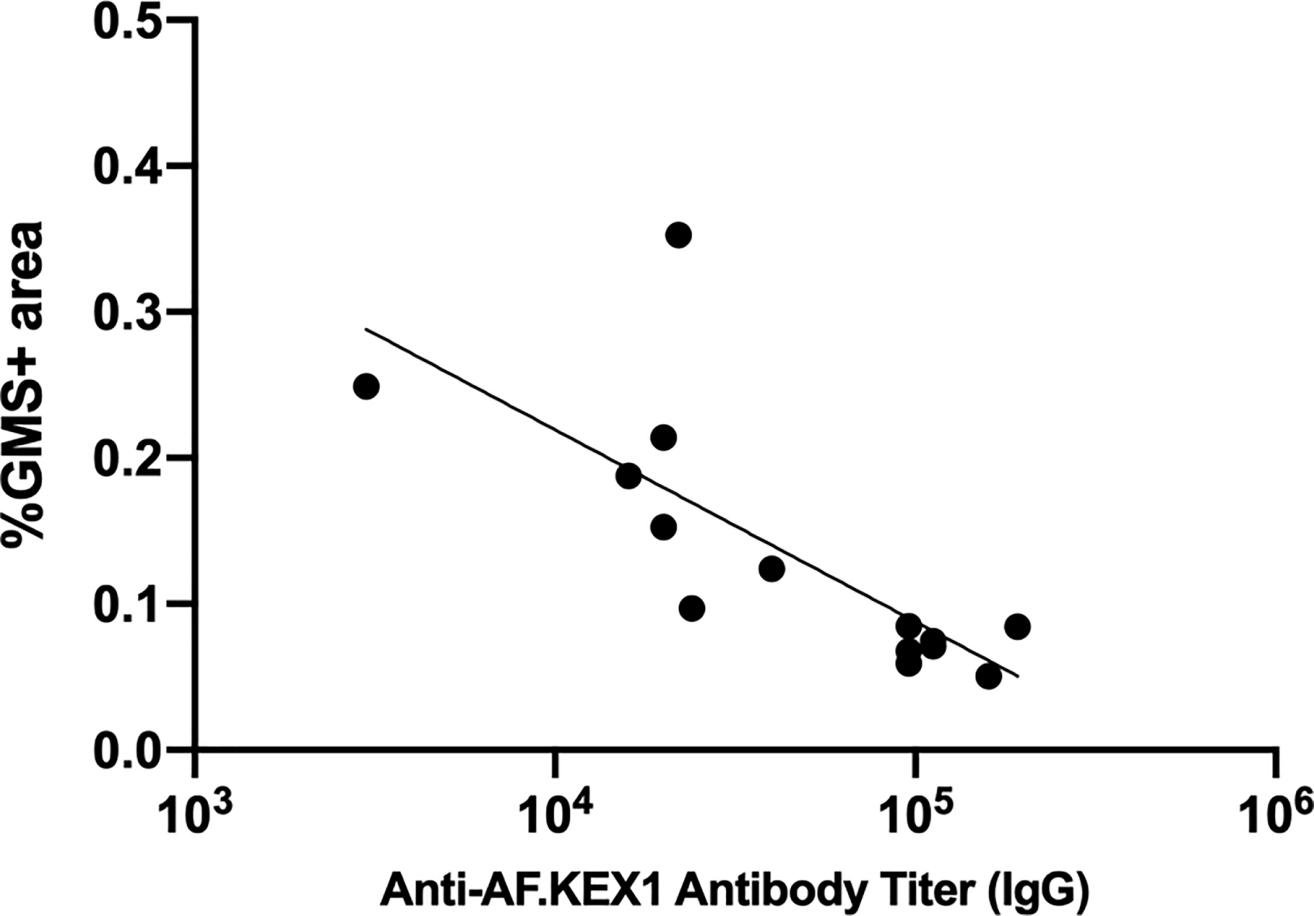
Correlation of anti-AF.KEX1 antibody titers with lung fungal burden. There was a significant negative correlation between peak antibody titer and fungal burden, by Spearman correlation (r=-0.8284, *p* < .001).

We evaluated the relationship between vaccine-induced AF.KEX1 IgG response and clearance of *Aspergillus* (**Figure 6**). We found that there was a significant negative correlation between the peak AF.KEX1 IgG titer and fungal burden (r=-.8284, *p*<.001) by Spearman.

## Discussion

This study examined the immunogenicity and protective efficacy of a recombinant *Aspergillus fumigatus* protein AF.KEX1 in two murine models of invasive pulmonary aspergillosis (IPA). Our results build upon previous research utilizing recombinant KEX1 protein as a vaccine candidate to achieve protection against *Pneumocystis* challenge (9). In the present studies, vaccination with AF.KEX1 protein and adjuvant generated a robust antibody response that was associated with significantly lower mortality and fungal burden in the lungs. These findings support AF.KEX1 as a potential vaccine candidate to prevent the development of IPA in individuals treated with immunosuppressive therapies.

Our results further indicate that a single immunization with AF.KEX1 and the squalene:water adjuvant Titermax is sufficient to establish protective levels of AF.KEX1 antibodies as the titers at the start of immunosuppression are not significantly different following one or two immunizations. This immunization regimen in mice generate a mixed humoral response based on IgG isotype evaluation. By both qPCR and histological quantification, fungal burden was significantly reduced in the lungs of mice vaccinated with AF.KEX1. Finally, higher antibody titers at the start of immunosuppression were also significantly correlated with the reduced terminal fungal burden determined by %GMS+ area. This supports the relationship between humoral responses to AF.KEX1 and protection against *A. fumigatus* challenge. Although there is an inverse correlation between IgG titers and organism burden, we have not directly demonstrated the protective role of anti-AF.KEX1 in mediating the protective efficacy. Studies to further delineate the role of T and B cells in the protection induced by AF.KEX1 are underway.

These results add to previous work in the field with recombinant protein vaccines and *Aspergillus* challenge, although much of this work has been conducted with known allergens, including Asp f3. The levels of protection associated with AF.KEX1 immunization reported here (75-100%) are similar to the levels of protection achieved with Asp f3 and TiterMax immunization (70-90%) (10, 15, 16). As seen with AF.KEX1 immunization, antibody levels to certain epitopes of Asp f3 are correlated with level of protection.

There are limitations to this study. The sample sizes were small and thus limited the depth of analysis. Only female mice were used in the corticosteroid cohort which limited sex comparison, though no differences in mortality or fungal burden by sex were observed in the tacrolimus/hydrocortisone immunosuppression model. Additionally, the mortality achieved with cortisone immunosuppression with *Aspergillus* strain Af293 challenge was notably less than that achieved by Ito et al. with the same immunosuppression regimen (83% vs. 24%) (10), the level of mortality was highly reproducible in our hands (3 independent infections). The difference in mortality rate reported by Ito et al. and that of the present study may be due to the use of a more virulent clinical *A. fumigatus* challenge strain (AFCOH1) compared to the laboratory isolate Af293 used here. Nevertheless, significant reduction in morbidity, mortality, and lung organism burden was demonstrated in both the cortisone model and the more severe tacrolimus/hydrocortisone model.

This is the first study to explore the role of AF.KEX1 protein in protection from invasive pulmonary aspergillosis and provides evidence for further exploration of this vaccine candidate. Future studies will determine if vaccination with AF.KEX1 is protective in additional models of other types of *Aspergillus* infections that are of clinical concern, including chronic pulmonary aspergillosis (17). In summary, vaccination with AF.KEX1 prior to immunosuppression could be an important strategy for prevention of IPA in at-risk individuals. In addition to successful prevention of infection, a successful vaccine strategy would be of benefit by providing an alternative to long term antifungal prophylaxis and a reduction of selection pressure in the development of azole-resistant *Aspergillus*.

## Data Availability Statement

The raw data supporting the conclusions of this article will be made available by the authors, without undue reservation.

## Ethics Statement

The animal study was reviewed and approved by University of Georgia Institutional Animal Care and Use Committee.

## Author Contributions

ER, WR, and KN conceived and planned the experiments. ER, SK, and BC performed the experiments and data analysis. MM and KN supervised the project and assisted in data interpretation. ER and KN wrote the manuscript in consultation with SK, BC, and MM. All authors contributed to the article and approved the submitted version.

## Funding

Work in the Norris laboratory was supported by the Georgia Research Alliance and the University of Georgia Research Foundation. Work in the Momany laboratory was supported by the USDA NIFA (MM; 2019-67017-29113) and the National Science Foundation (BC; DGE-1545433).

## Conflict of Interest

KN has a financial interest in NXT Biologics, Inc. and ER, WR, and KN are co-inventors of inventions related to research reported in the enclosed paper. An approved plan is in place with the University of Georgia for managing potential conflicts.

The remaining authors declare that the research was conducted in the absence of any commercial or financial relationships that could be construed as a potential conflict of interest.
